# Suppressed ABA signal transduction in the spike promotes sucrose use in the stem and reduces grain number in wheat under water stress

**DOI:** 10.1093/jxb/eraa380

**Published:** 2020-08-21

**Authors:** Zhen Zhang, Jing Huang, Yanmei Gao, Yang Liu, Jinpeng Li, Xiaonan Zhou, Chunsheng Yao, Zhimin Wang, Zhencai Sun, Yinghua Zhang

**Affiliations:** 1 College of Agronomy and Biotechnology, China Agricultural University, Beijing, China; 2 Engineering Technology Research Center for Agriculture in Low Plain Areas, Heibei Province, China; 3 University of Essex, UK

**Keywords:** Endogenous hormones, grain number per spike, signal transduction, sucrose metabolism enzyme, transcriptome, water-soluble carbohydrates, water stress, wheat

## Abstract

Water stress is a primary trigger for reducing grain number per spike in wheat during the reproductive period. However, under stress conditions, the responses of plant organs and the interactions between them at the molecular and physiological levels remain unclear. In this study, when water stress occurred at the young microspore stage, RNA-seq data indicated that the spike had 970 differentially expressed genes, while the stem, comprising the two internodes below the spike (TIS), had 382. Abscisic acid (ABA) signal transduction genes were down-regulated by water stress in both these tissues, although to a greater extent in the TIS than in the spike. A reduction in sucrose was observed, and was accompanied by increases in cell wall invertase (CWIN) and sucrose:sucrose 1-fructosyl-transferase (1-SST) activities. Hexose and fructan were increased in the TIS but decreased in the spike. ABA was increased in the spike and TIS, and showed significant positive correlation with CWIN and 1-SST activities in the TIS. Overall, our results suggest that water stress induces the conversion of sucrose to hexose by CWIN, and to fructan by 1-SST, due to increased down-regulation of ABA signal transduction related-genes in the TIS; this leads to deficient sucrose supply to the spike and a decrease in grain number.

## Teaser

Deficient sucrose and weak expression of ABA signal transduction genes in the spike may be the major reasons for the decrease in grain number under water stress.

## Introduction

Wheat (*Triticum aestivum*) is a major food crop and, as such, improvements in yield are required to meet the demands of the world’s growing human population, which is estimated to reach 9.4 billion by 2050 (Foulkes *et al.*, 2011). Coupled with this, water shortages are becoming increasingly common and represent a major limiting factor to crop growth (Rampino *et al.* 2006; Trenberth *et al.*, 2014). For example, in the North China Plain, rainfall only meets 25–40% of the water requirement of winter wheat; as a result, water shortages have become one of the most serious abiotic stressors for yield (Fang *et al.*, 2010; Liu *et al.*, 2016). Stress occurring during the reproductive period generally results in a greater effect on grain number than on grain weight within spikes (Fischer, 2008, 2011), as it is far more regulative and plastic than grain size (Sadras and Slafer, 2012). Hence, it seems likely that improvements in grain yield may derive from improvements in grain number, particularly under water stress conditions.

Generally, grain number depends on the number of fertile florets at anthesis (Borràs-Gelonch *et al.*, 2012; Ferrante *et al.*, 2013), which is mainly determined during the stem elongation period from the terminal spikelet to anthesis stages (e.g. González *et al.*, 2003, 2005; Guo *et al.*, 2018). This can be divided into a period of generation of floret primordia (from the terminal spikelet stage to the young microspore stage, YM) and a period of floret abortion (from the YM stage to anthesis). The spikelet usually achieves the maximum number of floret primordia at the YM stage even under stress conditions, such as shading (Fischer and Stockman, 1980), trimming of the spike (Serrago *et al.*, 2008), shortened photoperiod (Bancal, 2008), low nitrogen (Ferrante *et al.*, 2010), and delayed sowing (Zhu *et al.*, 2019). Reductions in grain number are greater when stress occurs at the YM stage, rather than either before or after it, and supply of assimilates to the anthers is important at this time (Ji *et al.*, 2010). During the YM stage, wheat is characterized by rapid growth of the stem (mainly in the two internodes below the spike, TIS) and spike, and by floret abortion. Hence a number of studies have examined the allocation of assimilates between these organs, and there is evidence that floret abortion is due to the weaker competive ability of the spike compared to the stem (e.g. Kirby, 1988; Foulkes *et al.*, 2011; González *et al.*, 2011). The flag leaf has generally been recognized as a major contributor of photoassimilates to the developing stem and spike, and to final grain set during this period (e.g. Simpson *et al.*, 1983; Saini *et al.*, 1984; Lopes *et al.*, 2006; Carmo-Silva *et al.*, 2017). However, the mechanisms by which the spike, the TIS, and flag leaf respond to stress and coordinate assimilate allocation with each other have not been clarified.

The identification of genes responsible for stress tolerance facilitates the genetic improvement of crops through marker-assisted selection or gene transformation. The transcriptional responses of plant organs to drought during the reproductive stage have been studied in barley (Guo *et al.*, 2009) and Bermuda grass (Shi *et al.*, 2015). Based on studies of the transcriptome, roots are known to show a stronger transcriptomic response to stress compared to leaves in soybean (Fan *et al.*, 2013), chickpea (Wang *et al.*, 2012), rice (Minh-Thu *et al.*, 2013), horse gram (Bhardwaj *et al.*, 2013), and *Jatropha curcas* (Sapeta *et al.*, 2016). In wheat, there is a need to identify the responses to stress at the transcriptional level in the spike, TIS, and flag leaf.

A significant positive relationship has been reported between spike dry matter accumulation (DMA) and the number of fertile florets in wheat (Fischer, 1985, 2011; González *et al.*, 2011). Spike DMA is a function of spike growth rate and duration, which are determined by assimilates partitioning to the spike (Fischer, 2011; García *et al.*, 2014). In this regard, dry matter partitioning (DMP) to the spike at the expense of the stem could be increased to favor spike DMA, and hence ultimately to increase the number of grains (Kirby, 1988). Overall, it seems that the fate of floral primordia is mostly determined by DMA, and by DMP between the flag leaf, the TIS, and the spike, and this ultimately affects grain number at maturity.

Plants respond to water stress through complex physiological and metabolic processes, which ultimately result in different DMA and DMP between organs (Lipiec *et al.*, 2013). In wheat, stored water-soluble carbohydrates (WSCs), composed mainly of sucrose, fructans, glucose, and fructose, are important indicators of assimilate accumulation. Variations in the concentrations of these WSCs in wheat are one of the valid factors influencing grain number and yield in water-limited environments (Xue *et al.*, 2008). Fructans are the major stored carbohydrates and function to protect against the negative effects of water stress (Garcia *et al.*, 2011). Sucrose is enzymatically hydrolysed into hexose and fructan to support the growth of sinks, such as developing seeds, fruits, and roots (Ruan, 2014). These metabolic processes are regulated by many enzymes and plant hormones (Albacete *et al.*, 2008; Harb *et al.*, 2010; Du *et al.*, 2013). For example, abscisic acid (ABA) and indole acetic acid (IAA) are known to regulate plant adaptive responses to various environmental stresses, and to affect diverse physiological and developmental processes (Ghanem *et al.*, 2011; Mohammad *et al.*, 2017). However, the precise metabolic regulation of sugars, enzymes, and hormones that coordinate DMA and DMP between the flag leaf, the TIS, and the spike under stress remains unclear.

The aim of this study was to determine how the responses and interactions of the flag leaf, TIS, and spike to water stress at the physiological, metabolic, and transcriptional level ultimately affect grain number in wheat. Our results provide insights into inter-organ coordination during water stress in this important crop.

## Materials and methods

### Plant growth and water conditions

**Fig. 1.**
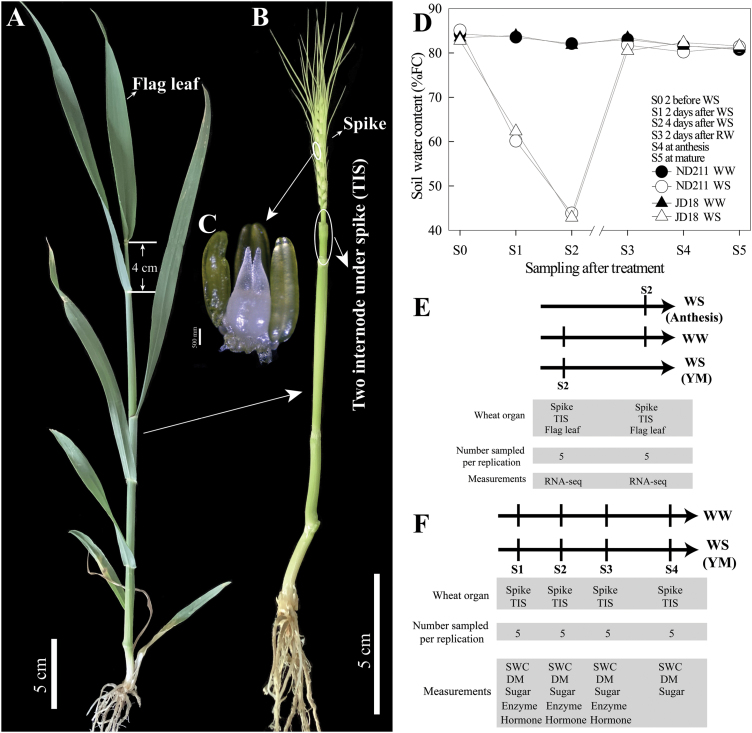
Schematic representation of the experiment and sample collection. (A) Auricle distance (AD=4 cm) measurements were used to identify the young microspore stage (YM) at the morphological level. (B) All leaf and sheath of wheat in (A) were removed. The position of TIS (two-internode under spike) is shown. (C) The first floret at central spikelet was observed, which corresponds to W7.5 stage, according to Waddington’s scale. (D) Soil water content was monitored from before treatment to mature stage. The letter S followed by a number indicates the sampling stage following different treatments. Data are shown from both cultivars. (E) Samples (WW and WS) for RNA-seq were collected at the 4 d after water treatment occurred at YM and anthesis stages, respectively. (F) Samples were collected at 2 d (S1), 4 d after WS (S2) and 2 d after re-watering (RW; S3), and then at anthesis (S4) when water treatments occurred at the YM stage. (This figure is available in colour at *JXB* online.)

The experiment was conducted at the Science Park of the China Agricultural University, Beijing, China (40.02°N, 116.28°E). Two cultivars of winter wheat (*Triticum aestivum*) were selected, namely ‘NongDa211 (‘ND211’) and ‘JingDong18’ (‘JD18’). Seeds were planted in pots (30 cm high, 27 cm diameter) filled with 9 kg soil that contained 0.3 g kg^–1^ urea (46% N), 0.75 g kg^–1^ superphosphate (20% P_2_O_5_), and 0.3 g kg^–1^ potassium sulfate (50% K_2_O). A total of 45 seeds were planted in each pot, and the seedlings were thinned to 30 per pot at the three-leaf stage. There were 24 pots for each cultivar and eight pots for each treatment. All pots were placed outside in the field and moved into a greenhouse by hand whenever there was rainfall. There were two watering treatments, which were controlled by weighing the pots: well-watered (WW), in which the soil water content was maintained at 80–85% of field water capacity, and water stress (WS), in which the soil water content was maintained at 40–45% of field water capacity. Soil water content (SWC) was measured as described by Sapeta *et al.* (2013). The water treatments were applied at the young microspore (YM) stage and at the anthesis stage. YM stage was determined as the day when the distance between the auricles of the flag leaf and the penultimate leaf was 4 cm (Oliver *et al.* 2005), and it is equivalent to the W7.5 stage (Waddington *et al.*, 1983; Fig. 1A–C). The WS treatments lasted 4 d, after which the pots were re-watered back to 80–85% of field water capacity (Fig. 1D). Measurements were only conducted on main stems, 20 of which were labeled in each pot early during development. At each sampling time-point, five main stems of equal growth status were collected per pot and pooled together, and three replicate pots were sampled. The sampling scheme is shown in Fig. 1E, F.

### RNA extraction and sequencing

Pooled samples of five spikes, TIS, and flag leaves were collected at 4 d after the beginning of treatment (S2) at the YM and anthesis stages. Total RNA was extracted using TRIzol Reagent (Tiangen, China) following the manufacturer’s instructions. RNA purity and integrity were assessed using an Agilent Bioanalyzer 2100 system (Agilent Technologies, Palo Alto, CA, USA). Paired-end sequencing libraries with an insert size of ~300 bp were sequenced using an Illumina HiSeq 4000 TM platform (Illumina, Inc., San Diego, CA, USA). Clean data (clean reads) were obtained by removing reads containing adapters, or poly-N (N>10%), and low-quality reads (>50% bases with quality scores≤ 5) from the raw data (Supplementary Table S1 at *JXB* online). FPKM values are fragments per kilobase of exon model per million mapped reads, according to Mortazavi *et al.* (2008), with paired-end reads. Genes were considered to be differentially expressed if they had a *q*-value <0.05 according to DESeq (Anders and Huber, 2010), and up-regulation of log_2_fold-change >0 or down-regulation of log_2_fold-change <0. Gene Ontology (GO) and Kyoto Encyclopedia of Genes and Genomes (KEGG) annotation and enrichment analyses were performed using GOSeq (v.1.22) and KO-Based Annotation System (KOBAS) (v.2.0), respectively, as described by Wei *et al.* (2015). To evaluate general trends of expression, the differentially expressed genes (DEGs) were subjected to *k*-means clustering analysis. We first clustered the DEGs using the *k*-means function in the R software, with *k*=4 within the cluster package via Euclidean distance.

### Quantitative real-time PCR

Quantitative real-time (qRT–PCR) was performed to test the results of RNA-seq using RNA extracted from independently grown and treated seedlings. Three randomly selected primer sequences were used for qRT–PCR, and the results are shown in Supplementary Table S2 and Fig. S1. Total RNA (extracted as described above) was reverse-transcribed into cDNA using a PrimeScript RT Reagent Kit (Takara, China). qRT–PCR reactions were visualized by *Taq* DNA Polymerase and SYBR Green fluorescent dye (Takara) on a QuantStudi 6 Flex System (ThermoFisher, China). The relative quantitative method was used to compare the expression difference of the target mRNA. The 2^–∆∆*C*T^ method was used to quantify the relative expression of the target gene mRNA.

### Sampling procedure and measurement of physiological parameters

Five fresh spikes and TIS were sampled at the YM stage at 2 d (S1) and 4 d (S2) after the beginning of treatment, at 2 d after rewatering (S3), and at anthesis (S4) (Fig. 1E, F). The tissues were immediately frozen in liquid nitrogen and then stored at −80 °C for subsequent extraction of hormones and enzymes (see below). Another five plants were sampled and divided into spikes, TIS, flag leaves, and other organs, and then dried (at 80 °C for 48 h) to a constant weight for determination of dry matter accumulation (DMA, g), dry matter partitioning (DMP, %; dry weight of organ versus dry weight of whole plant), and dry matter ratio between organs (%; for example, weight of spike versus weight of TIS). These dried tissues were then used for measurement of sugars. At maturity, twenty spikes per pot were sampled to determine the grain yield per spike (total grain weight per spike), grain number per spike, and t1000-grain weight. In addition, the effects of grain position within the spike were examined. The spikelets within a spike were numbered, with position 1 being the basal spikelet. We then recorded the number of superior grains (first and second florets) and inferior grains (third and fourth florets) within each spikelet.

### Measurement of sugars

Sugars were extracted according to Wardlaw and Willenbrink (1994). In brief, 200 mg tissue samples were extracted directly in 10 ml boiling water for 1 h, the supernatant was collected, and the residues were extracted a second time in 7 ml boiling water for 1 h. The extract was then passed through a 0.45 μm filter membrane, and 20 μl was used for measurements by HPLC (Waters, Milford, MA, USA). The separation was carried out on an Ultimate^TM^-NH_2_ column (Waters) at 25 °C with an acetonitrile:water mixture (80:20 v/v; 1 μl min^–1^). The chromatographic peak area was determined with a refractive index detector (2420; Waters) to calculate the glucose, fructose, and sucrose concentrations. Standard solutions (1% v/v) of glucose, fructose, and sucrose were used for determination of the concentration of fructan.

### Activity assays for invertase, 1-fructan exohydrolase, and sucrose:sucrose 1-fructosyl-transferase

Invertase extracts were prepared according to Zinselmeier *et al.* (1999). Tissue samples of 200 mg were ground in liquid nitrogen using a mortar and pestle, and extracted twice in 1.5 ml of buffer consisting of 50 mM HEPES-NaOH (pH 7.2), 5.0 mM MgCl_2_, 15% (v/v) ethylene glycol, 1.0 mM EDTA, and 1.0 mM DTT (1 M NaCl was also added when extracting insoluble acid invertase). The extracts were combined, centrifuged at 14 000 *g* for 5 min at 4 °C, and the supernant was used for determination of soluble vacuolar invertase (VIN) activity. The precipitate was resuspended in 1.5 ml of extraction buffer for the determination of cell wall invertase (CWIN) activity. Enzyme activities were determined using a spectrophotometer, according to Shen *et al.* (2018).

For determination of the activities of 1-fructan exohydrolase (1-FEH) and sucrose:sucrose 1-fructosyl-transferase (1-SST), tissue samples of 0.5 g were fully ground in liquid nitrogen and homogenized in 0.05 M McIlvaine buffer (1:1, w/v, pH 5.5), which contained 2 mM EDTA, 2 mM β-mercaptoethanol, 5 mM ascorbic acid, and 10% PVPP (w/w), as described by Asega and Carvalho (2004). Proteins were precipitated with (NH_4_)_2_SO_4_ until 80% saturation was reached and then re-suspended in the extraction buffer at a ratio of ~10 g fresh mass per cm^3^.The proteins were then desalted by centrifugation in Bio-Gel P-6 DG (BioRad, USA) with the same buffer. The desalted extracts were used for protein measurements and determination of enzymatic activities by the reducing sugar detection method for 1-FEH, using fructose (Sigma–Aldrich Co., USA) as standard, and using the external standard method for 1-SST, as described by Portes and Carvalho (2006). Incubation times were 30 min for 1-FEH and 1 h for 1-SST.

### Extraction and quantification of ABA and IAA

Tissue samples of 0.5 g were extracted for ABA assays according to Liu *et al.* (2011). The samples were thoroughly mixed with 5 ml of 80% (v/v) methanol containing 1 mmol l^–1^ butylated hydroxytoluene, and the solution was passed through a Chromosep C18 column (C18Sep-Park Cartridge, Waters). The fractions were vacuum-dried at 40 °C and dissolved in 1 ml of phosphate-buffered saline that contained 0.1% (v/v) Tween 20 and 0.1% (w/v) gelatin (pH 7.5). The ABA and IAA concentrations were determined as previously described (Liu *et al.*, 2011), by the Phytohormones Research Institute, China Agricultural University, using an ELISA Reader (model EL310, Bio-TEK, Winooski, VT), and following the manufacturer’s instructions. The recovery rate for ABA and IAA was 89.5±3.2%.

### Statistical analysis

All data were analysed using the SPSS software (v. 17.0), RStudio (v. 1.2.5019), and R (v. 3.6.1). Differences between treatment means were identified using the LSD test at *P*≤0.05 level.

## Results

### Water stress at the young microspore stage results in a greater reduction in grain number than at anthesis

Compared with the well-watered (WW) control, there were significant decreases (*P*<0.05, LSD) in grain yield per spike in both varieties (‘ND211’ and ‘JD18’) when water stress (WS) was applied, at either the young microspore (YM) stage or at anthesis, and the effect was greater at the YM stage (Table 1). This was despite the fact that 1000-grain weight (TGW) was significantly increased (*P*<0.05, LSD) by WS at the YM stage, and also showed an increase (although non-significant) as a result of WS at anthesis. The effect on grain yield was thus attributable to decreases in grain number per spike, which was greater when WS was applied at the YM stage than at anthesis. Hence the YM stage appeared to be more sensitive to water stress in terms of reduction in grain number.

**Table 1.** Grain yield per spike and yield components of winter wheat under well-watered (WW) and water stress (WS) conditions at the young microspore (YM) and anthesis (A) stages. Values are the mean ±SD of three biological replicates. n=20. Letters indicate significant differences between treatments (*P* < 0.05, LSD).

Cultivar	Treatment	Grain number per spikes			1,000-grain weight (g)	Grain yield (g spike^-1^)
		Superior grains	Inferior grains	Total grains		
*ND211*	WW	29.4±1.1a	8.8±0.5a	38.2±0.3a	38.8±0.6b	1.5±0.5a
	WS(YM)	15.8±1.5c	1.0±0.8c	16.8±1.2c	44.4±1.2a	0.8±0.1c
	WS(A)	22.4±1.3b	2.3±0.7b	24.7±0.8b	40.7±0.8b	1.2±0.3b
*JD18*	WW	32.1±0.8a	8.5±0.5a	40.6±0.6b	37.1±1.1b	1.6±0.8a
	WS(YM)	18.3±1.0c	1.9±0.6c	20.2±0.9c	45.1±0.7a	1.0±0.4c
	WS(A)	27.7±1.5b	3.3±1.2b	31.0±0.4b	39.5±0.7b	1.4±0.6b
	S.E.	1.5	0.7	2.2	2.9	0.2

† Values followed by the same letter in a column are not significantly different according to LSD (0.05).

### Effects of water stress at the young microspore stage on grains at different positions within the spike

**Fig. 2.**
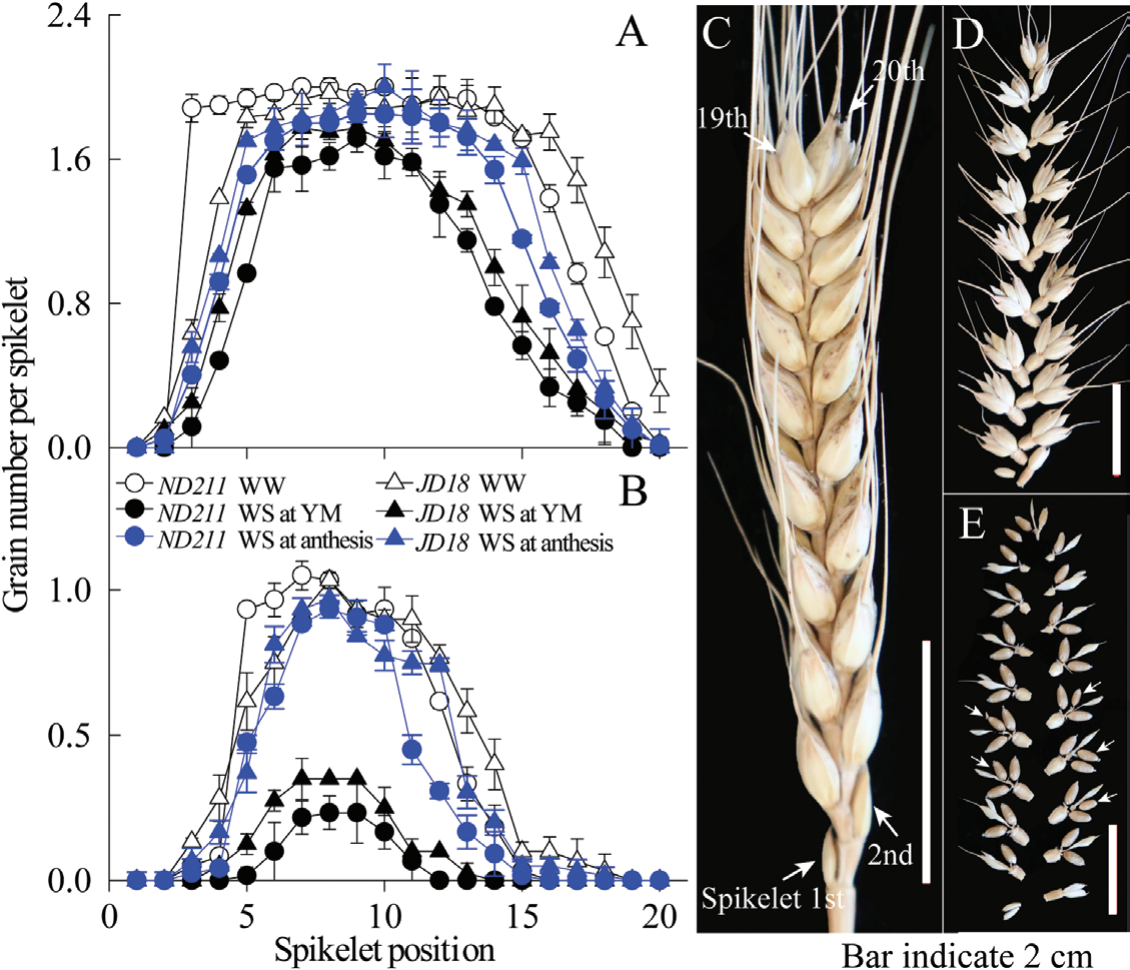
Effect of well-watered (WW) treatments and water stress (WS) at the young microspore (YM) stage and anthesis, on the SGN (the first and second grain within a spikelet; A) and IGN (the third and fourth grain within a spikelet; B) at different spikelet positions in the *ND211* and *JD18* cultivars. Bars represent the standard error (n=20). The spikelets were numbered from bottom to top, with the first spikelet at the bottom of the spike identified as 1; moreover, 1-6, 7-14 and 15-20 spikelets were basal, central and apical spikelets, respectively (C). Correspondingly, each spikelet was separated (D) and then divided into grains (E). Arrows indicate inferior grains and others are superior grains (E). (This figure is available in colour at *JXB* online.)

To explore and identify the specific variation in grain number when WS occurred at YM and anthesis stages, grain number was divided into two classes, superior grain number (SGN, first and second florets) and inferior grain number (IGN, the third and fourth florets), at different spikelet positions within a spike (Fig. 2C-E). SGN and IGN in the central (7–14) spikelet was higher than the apical (15–20) and basal (1–6) spikelet for the two cultivars ‘ND211’ and ‘JD18’ (Fig. 2A, B). The IGN at the apical and basal spikelet was almost completely aborted under WS conditions (Fig. 2B). The SGN of the apical spikelet and the IGN of the central spikelet were reduced for both cultivars under WS compared with WW controls, and the reduction was higher under WS at the YM stage than at anthesis (Fig. 2A, B). These results suggest that WS reduced the IGN at the central spikelet, and the SGN and IGN at the apical and basal spikelet, respectively for the two cultivars.

### DEGs increase when water stress occurs at YM than at anthesis

**Fig. 3.**
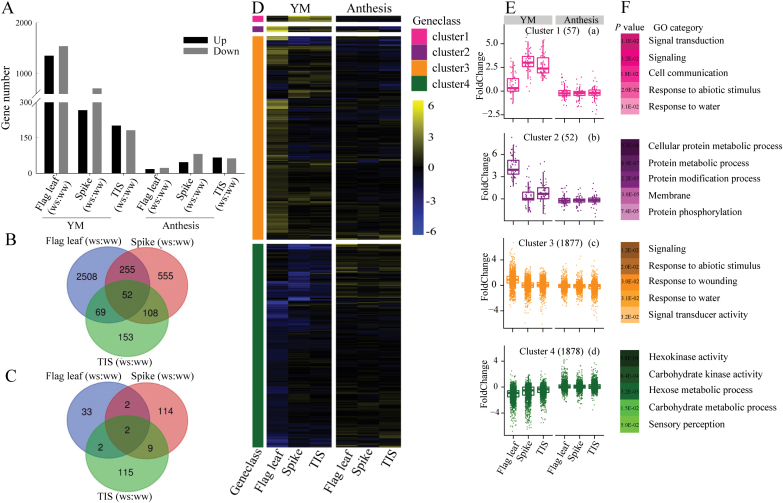
Analysis of RNA-sequencing data of the spike, two-internode under spike (TIS) and flag leaf during young microspore (YM) and anthesis stages under water stress (WS) and well-watered (WW) conditions. (A) The numbers of the differentially expressed genes (DEGs; WS versus WW) for spike, the TIS and flag leaf during the YM and anthesis stages. (B) Venn diagram analysis of all DEGs of each organ during the YM stage. (C) Venn diagram analysis of all DEGs of each organ during the anthesis stage. (D) *k*-means clustering of DEGs. Expression distribution of genes activated or repressed 4 d after water stress treatment. The number indicates log_2_ ratio of the fold-change between the WS groups with the respective control group for the spike, TIS and flag leaf at the YM and anthesis, respectively. Yellow color represents genes that have higher expression levels after water stress and blue indicates reduced expression. (E) a-d, expression patterns of genes specific to each organ. (F) The GO enrichment analysis of genes in each cluster, with *P* values and GO category. (This figure is available in colour at *JXB* online.)

To unravel the transcriptional response of these organs to WS at YM stage and anthesis, we performed RNA-seq analysis for the spike, the TIS and flag leaf in the ‘ND211’ cultivar. We then performed qRT–PCR analyses to verify our RNA-seq data. As shown in Supplementary Table S2 and Fig. S1, three randomly selected genes including *Ta*GA2ox (encoding gibberellin oxidase), *Ta*NIT4 (encoding nitrate assimilatory enzymes), and *Ta*1-SST (encoding sucrose:sucrose 1-fructosyl-transferase) showed similar expression patterns in qRT–PCR compared to those quantified by RNA-seq. Pairwise measurements (WS versus WW) of differentially expressed genes (DEGs) showed that the spike, TIS and flag leaf had more DEGs, particularly down-regulated DEGs, at YM stage than at the anthesis stage (Fig. 3A; Supplementary Table S3-4). Moreover, different organs showed opposite responses to WS between the two stages. For instance, the flag leaf had 2884 DEGs, while TIS had the lowest DEGs (382) when WS occurred at YM stage (Fig. 3B). In contrast, the flag leaf had the lowest number (39) of DEGs, while the spike and the TIS maintained the highest number of DEGs (~127) when WS occurred at the anthesis stage (Fig. 3C). Further analysis showed that only the TIS consistently showed similar up-regulated DEGs (201, 181) and down-regulated DEGs (66, 62) at the two stages, respectively (Fig. 3A). The spike and flag leaf consistently had more down-regulated DEGs than up-regulated DEGs at both stages.

A closer examination of the DEGs found among these three organs when WS occurred, revealed 52 common DEGs, and two common DEGs, at YM stage and anthesis, respectively (Fig. 3B, C). Compared with WS at YM stage, during WS at anthesis, DEGs were reduced by 98.7% in flag leaf, 79.5% in spike and 24.8% in TIS. These findings suggest that the three organs displayed more DEGs under WS at the YM stage than at anthesis; however, the change in TIS was lower than that in the spike and flag leaf.

### Cluster analysis and prediction of function of DEGs

To identify the general trend of gene expression profiles, we applied hierarchical clustering to all 3864 DEGs between WW and WS treatments to the *k*-means (*k*=4) analysis, which resulted in four large clusters (Supplementary Table S5-8). These four clusters were then visualized with heat map and centroid views (Fig. 3D, E) and then annotated using singular enrichment analysis using the GOseq tool (Young *et al*., 2010; Fig. 3F; Supplementary Table S9).

Clusters 1 contained genes with an obviously higher expression in the spike, TIS and flag leaf, as induced by WS at the YM stage than that at the anthesis stage. Genes only in the spike showed an obviously high expression in cluster 3 at the YM stage.These genes were mainly involved in signal transduction in response to water and abiotic stimuli [Fig. 3E(a), E(c)]. In cluster 2, the genes were involved in protein metabolic processes [Fig. 3E(b)]. Genes involved in carbohydrate metabolic processes were enriched in cluster 4, and showed lower expression when WS was applied at YM stage than at anthesis, for spike, TIS and flag leaf [Fig. 3E(d)].

### Water stress induces hormone signaling and sucrose metabolism

**Fig. 4.**
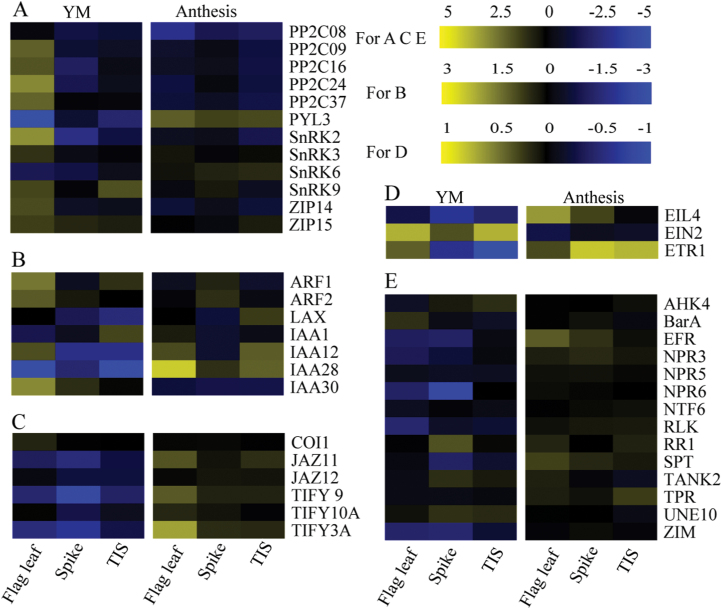
Expression of plant hormone signal transduction (KEGG) genes in the spike, TIS and flag leaf affected by water stress occurred at the YM and anthesis stage, respectively. (A) Expression heat maps for relative expression of ABA metabolism-related genes. (B) Expression heat maps for relative expression of IAA metabolism-related genes. (C) Expression heat maps for relative expression of jasmonic acid (JA) metabolism-related genes. (D) Expression heat maps for relative expression of ethylene metabolism-related genes. (E) Expression heat maps for relative expression of other genes responsive to stress. The relative expression levels (log_2_) of genes above 0 represent up-regulation, whereas those below 0 represent down-regulation. (This figure is available in colour at *JXB* online.)

Given that the signal transduction pathway and carbohydrate metabolism-associated genes were mainly induced as described in Fig. 3D-F and Supplementary Table S10, their roles were further explored. Among DEGs, there were 12 ABA-, seven IAA-, six jasmonic acid (JA)- and three ethylene- and other hormone-response genes annotated in their respective pathways (Fig. 4). These genes were mainly down-regulated under WS at YM stage but up-regulated under WS at anthesis, including genes in the JA pathway. ABA and IAA are important stress-inducible hormones (Rahman, 2013; Mohammad *et al.*, 2017). Thus, genes related to ABA and IAA signal transduction were mainly analysed (Fig. 4A, B), and genes related to jasmonic acid, ethylene and other hormones (Fig. 4C, D) have not been described in detail. Interestingly, most of the genes related to ABA signal transduction were only up-regulated in flag leaf, but down-regulated in the TIS and spike under WS at YM stage. Moreover, spike displayed lower gene expression than TIS, such as in a group of protein phosphatase 2Cs (PP2C). The TIS and spike maintained similar expression of IAA signal transduction genes, such as auxin response factors (ARFs). These results suggested that ABA and IAA signal transduction related-DEGs were up-regulated only in the flag leaf, while the TIS showed greater involvement in ABA signal transduction than the spike as WS occurred at YM stage.

**Fig. 5.**
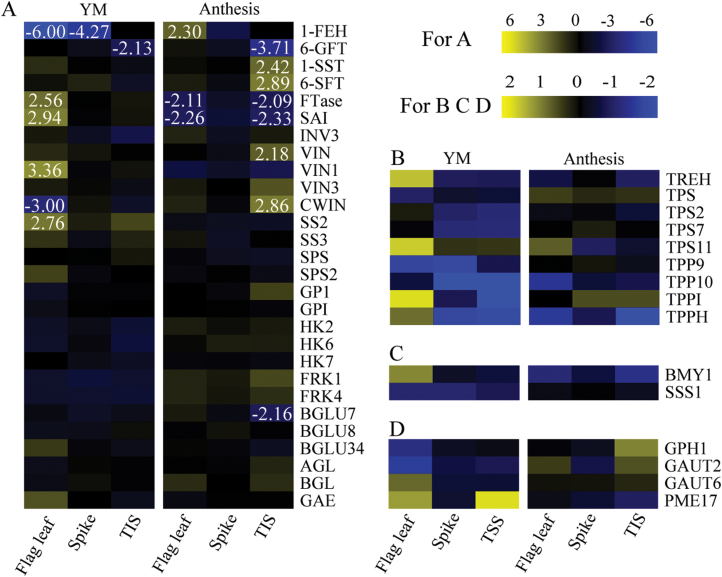
Expression of starch and sucrose metabolism (KEGG) genes in the spike, TIS and flag leaf affected by water stress occurred at the YM and anthesis stage, respectively. (A) Expression heat maps for relative expression of sucrose metabolism-related genes. (B) Expression heat maps for relative expression of trehalose metabolism- related genes. (C) Expression heat maps for relative expression of starch metabolism-related genes. (D) Expression heat maps for relative expression of other metabolism-related genes. The relative expression levels (log_2_) above 0 represent up-regulation, whereas those below 0 represent down-regulation. (This figure is available in colour at *JXB* online.)

The number of DEGs involved in sucrose, trehalose, starch and other metabolism were 28, nine, two and four, respectively (Fig. 5; Supplemental Table S10). All these DEGs involved in starch and sucrose metabolism pathway were collected from different organs under different stages. For example, genes encoding sucrose synthase were defined as DEGs only in flag leaf at the YM stage. Genes encoding trehalose-phosphatase were defined as DEGs only in the spike at the YM stage. Thus, the two DEGs were collected. Moreover, expression of the two DEGs in other treatments are also presented in the heatmap. Among these different metabolic pathways, sucrose metabolism had the most DEGs and was the major factor regulating seed and fruit set (Ruan *et al.*, 2012). Thus, genes in sucrose metabolism were further analysed and classified into three groups to identify individual gene expression patterns. The first group included genes whose expression was up-regulated at least two-fold, as defined by Zhu *et al.* (2011); genes in this group with higher expression levels were mainly responsible for the conversion of sucrose to hexose in the flag leaf under WS at YM stage, and to fructan in the TIS under WS at anthesis. Therefore, sucrose hydrolysis may be an indicator of a response to WS. Genes in the second group were characterized by at least two-fold down-regulation of gene expression; these genes in the flag leaf and the TIS were involved in sucrose hydrolysis under WS at anthesis, and fructan exohydrolase (1-FEH) was found in the flag leaf and spike under WS at YM stage. The third group was defined by gene expression changes within two-fold and always at low levels; this group of genes may not be crucial in determining sucrose metabolism.

### TIS maintains steady dry matter partitioning when water stress occurs at young microspore stage

**Fig. 6.**
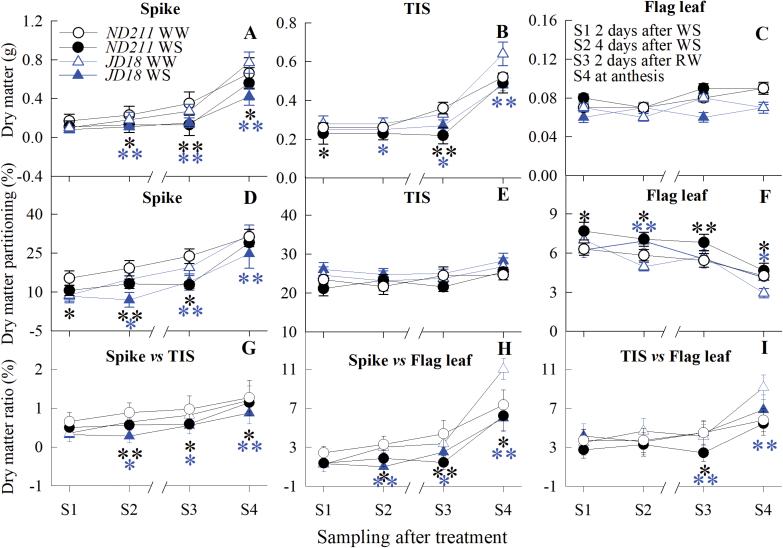
Effect of water stress (WS) and well-watered (WW) treatments at the young microspore (YM) stage, on the dry matter weight (A-C) and partitioning (D-F) in the spike, two-internode under spike (TIS) and flag leaf of the ‘ND211’ and ‘JD18’ cultivars, and their dry matter ratios (G-I, spike versus TIS, spike versus flag leaf, TIS versus flag leaf, respectively). S1, 2 d after WS; S2, 4 d after WS; S3, 2 d after re-watering; S4, at anthesis. Significant differences between the WS and WW treatments are indicated by asterisks (*P<0.05 and **P<0.01, Student’s *t*-test). Asterisks above or below the line represent values at the WS treatment that were significantly higher or lower than corresponding WW treatment. Bars represent standard error (n=5).

Dry matter accumulation (DMA) and partitioning (DMP) between the flag leaf, spike and TIS were tested from the YM to anthesis stages. The DMA of flag leaf, spike and TIS continued to increase before anthesis for the two cultivars (Fig. 6A-C). The DMA of the spike and the TIS was repressed from the beginning of WS occurring at the YM stage when compared with WW controls, while the flag leaf did not show any difference. The DMP in the flag leaf decreased gradually, but increased in the TIS and spike from S1 (2 d after WS) to S4 (at anthesis; Fig. 6D–F). Compared with WW controls, the DMP in the flag leaf increased markedly, while it decreased in the spike under WS for the two cultivars (P < 0.05, Student's *t*-test). The DMP of the TIS was not affected by WS. The DMA ratios between the spike versus TIS, spike versus flag leaf, and TIS versus flag leaf, decreased in response to WS, when compared with WW controls (Fig. 6G-I). Collectively, these results indicated that WS repressed the competitive capacity of the spike for DMP relative to TIS, which in turn resulted in poor spike fertility, in both cultivars tested.

### Changes in hexose and fructan concentrations in TIS and spike under water stress

**Fig. 7.**
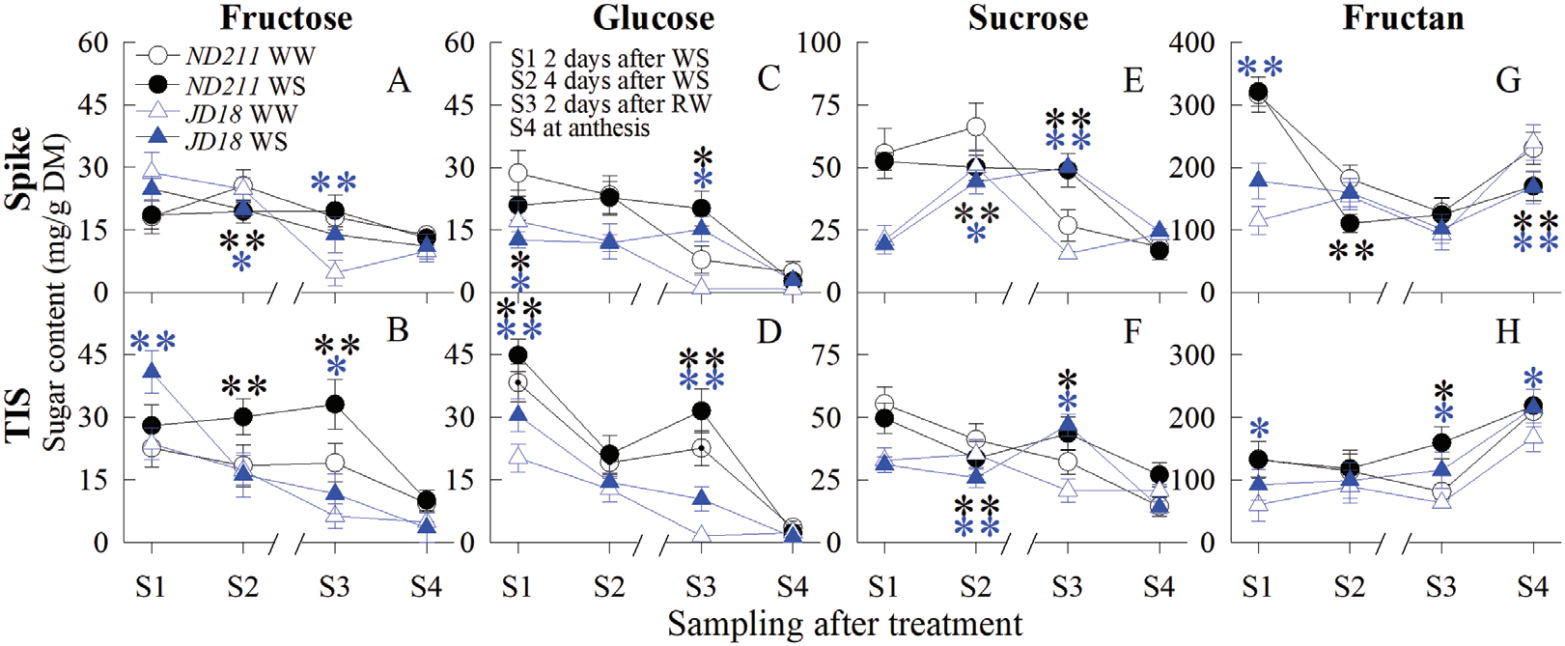
Effect of water stress (WS) and well-watered (WW) treatments at the young microspore stage, on fructose (A, B), glucose (C, D), sucrose (E, F), and fructan (G, H) concentrations in the spike and two-internode under spike (TIS) of the ‘ND211’ and ‘JD18’ cultivars. S1, 2 d after WS; S2, 4 d after WS; S3, 2 d after re-watering; S4, at anthesis. Significant differences between the WS and WW treatments are indicated by asterisks (*P<0.05 and **P<0.01, Student’s *t*-test). Asterisks above or below the line represent the value at the WS treatment that was significantly higher or lower than the corresponding WW treatment. Bars represent standard error (n=5). (This figure is available in colour at *JXB* online.)

It was clear that the DMA of the flag leaf was almost unaffected by WS at YM, and the increased DMP of the flag leaf was due to decreased DMA of the other two organs. Thus, four main sugars were only analysed in the spike and the TIS. As shown in Fig. 7 sucrose and hexose (glucose and fructose) decreased gradually until anthesis (S4), whereas fructan first decreased and then increased after re-watering. Hexose increased notably in the TIS but decreased in the spike for both cultivars under WS (Fig. 7A-D). WS resulted in a significant reduction of sucrose in the TIS and spike (Fig. 7E, F). Fructan was significantly induced in the TIS, while it was repressed in the spike (Fig. 7G, H). In the spike, ‘ND211’ had higher glucose, sucrose and fructan than ‘JD18’. In the TIS, only glucose in ‘ND211’ was higher than ‘JD18’.

### Activities of enzymes involved in sucrose metabolism change under water stress

**Fig. 8.**
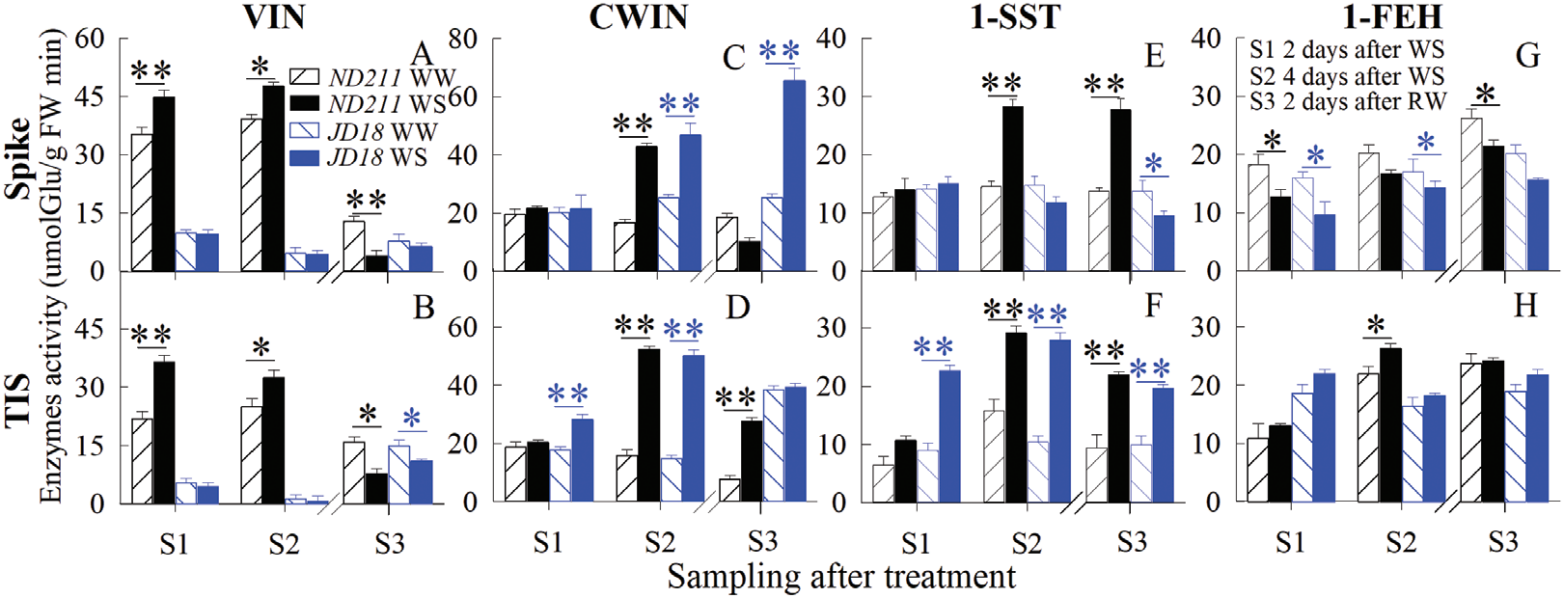
Effect of water stress (WS) and well-watered (WW) treatments at the young microspore stage on VIN (A, B), CWIN (C, D), 1-SST (E, F), and 1-FEH (G, H) activities in the spike and two-internode under spike (TIS) of the ‘ND211’ and ‘JD18’ cultivars. S1, 2 d after WS; S2, 4 d after WS; S3, 2 d after re-watering. Significant differences between the WS and WW treatments are indicated by asterisks (*P<0.05 and **P<0.01, , Student’s *t*-test). The break in the x-axis indicates the start of re-watering. Bars represent standard error (n=5). (This figure is available in colour at *JXB* online.)

Four major enzymes involved in sucrose metabolism were measured synchronously. The enzyme assay revealed that WS at YM stage had a positive effect on CWIN activity in the TIS and spike of both cultivars, but only on vacuolar invertase (VIN) activity in ‘ND211’ (Fig. 8A-D). The activity of 1-SST increased significantly in TIS of both cultivars, while it was induced only in the spike of ‘ND211’ (*P*<0.01, Student’s *t*-test) (Fig. 8E). Enzyme activity of 1-FEH was induced in the TIS of ‘ND211’, whereas it decreased in the spike of the two cultivars in response to WS, compared with WW controls (Fig. 8G, H). There was no obvious difference between the two cultivars regarding CWIN, 1-SST and 1-FEH activities, whereas only VIN in ‘ND211’ showed obvious higher activity than that in ‘JD18’.

### Water stress affects ABA and IAA concentrations

**Fig. 9.**
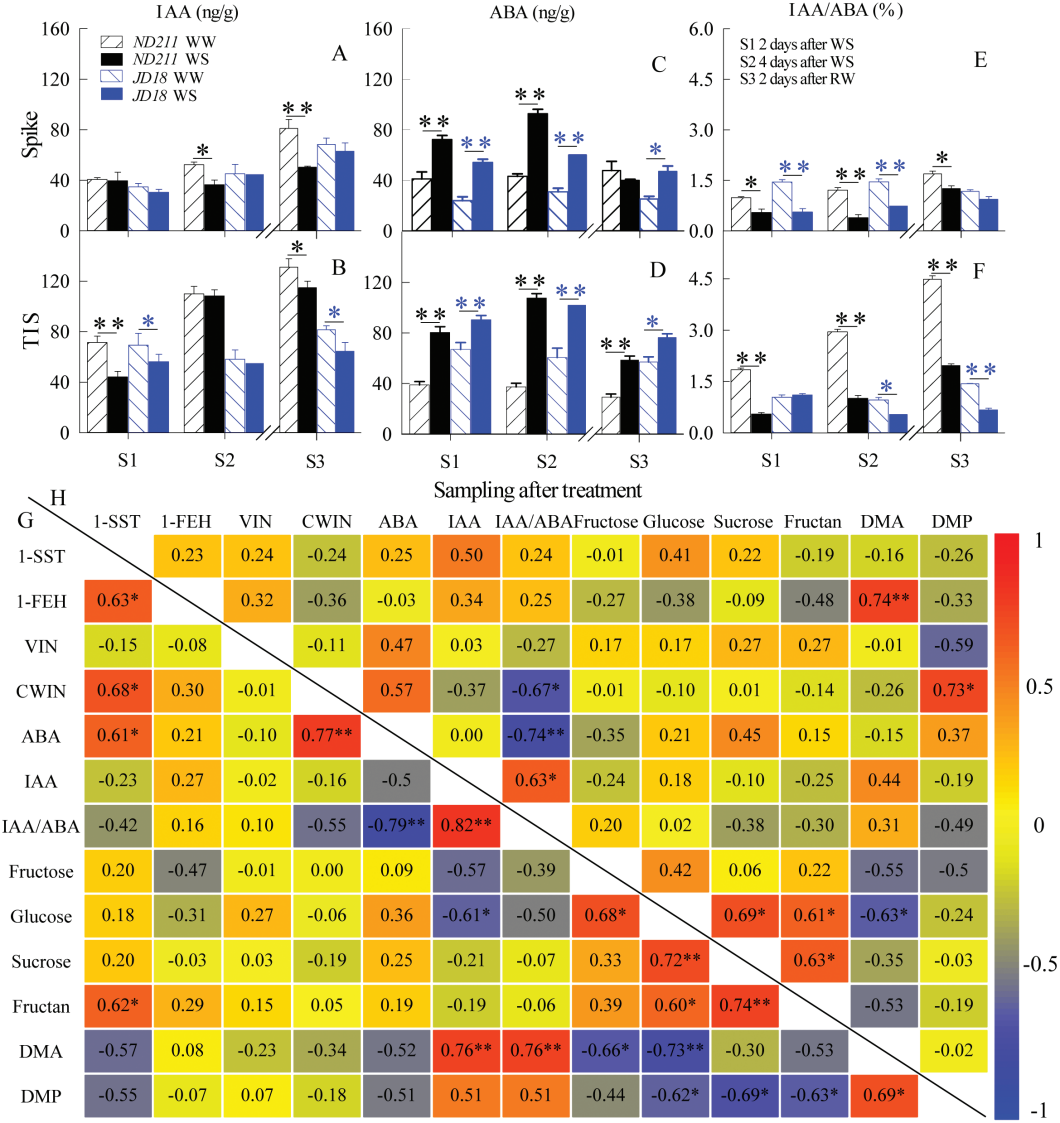
Effect of water stress (WS) and well-watered (WW) treatments at the young microspore stage on IAA, ABA concentrations and IAA/ABA ratio, and their correlation with sugars, enzymes and dry matter in the spike and two-internode under spike (TIS). Effect of WS on IAA (A, B), ABA (C, D) and IAA/ABA ratio (E, F) in the spike and TIS of the ‘ND211’ and ‘JD18’ cultivars. S1, 2 d after WS; S2, 4 d after WS; S3, 2 d after re-watering. Significant differences between the WS and WW treatments are indicated by asterisks (*P<0.05 and **P<0.01, Student’s *t*-test). The break in the x-axis indicates the start of re-watering. Bars represent stand error (n=5). (B) Correlation between enzymes, hormones, sugars and dry matter in the TIS (below the black line, G) and spike (above the black line, H) for the ‘ND211’ and ‘JD18’ cultivars (*P<0.05 and **P<0.01). The number in each box is the correlation coefficient. The colors reflect the changes in correlation coefficient: red color represents correlation coefficient with high and positive correlation and blue indicates high and negative correlation. (This figure is available in colour at *JXB* online.)

Growth regulation and grain set in cereals are mainly mediated by endogenous hormones (Albacete *et al.*, 2008). Moreover, transcriptional data showed that DEGs regulating ABA and IAA were primarily enriched among various hormone signal transduction pathways. Here, IAA and ABA concentrations were further measured in the same samples as those used for enzymatic analysis. Compared to WW, ABA was significantly induced (*P*<0.01, Student’s *t*-test), whereas IAA concentration was significantly decreased by WS at YM stage in the spike and TIS of both cultivars (*P*<0.05, Student’s *t*-test) (Fig. 9A-D). The IAA/ABA ratios in the spike and TIS were also repressed by WS (Fig. 9E-F).

In addition, the correlation between concentration of sugars, enzymes activity and hormone concentrations were analysed in the TIS and spike, respectively (Fig. 9G, H). In the spike, the IAA/ABA ratio significantly and negatively correlated with CWIN (Fig. 9H). In the TIS, the IAA/ABA ratio was significantly and positively correlated with DMA; ABA concentration was significantly and positively correlated with CWIN and 1-SST activity, IAA concentration was significantly and positively correlated with DMA, but negatively correlated with glucose; and 1-SST activity was significantly and positively correlated with 1-FEH activity, CWIN activity and fructan (Fig. 9G). In addition, glucose concentration was significantly and positively correlated with the other three sugars, and sucrose was significantly and positively correlated with fructan in both the spike and TIS (Fig. 9G, H).

## Discussion

### Increased sensitivity of wheat to water stress at young microspore stage

In this study, a significant reduction in grain yield was observed as water stress (WS) occurred at either the young microspore (YM) stage or anthesis, reflected by a significant decrease in grain number, despite an increase in the total grain weight (TGW, Table 1). This result is consistent with numerous reports that grain yield is more strongly linked with grain number rather than TGW (e.g. Shearman *et al.*, 2005; Sanchez-Garcia *et al.*, 2013; Lynch *et al.*, 2017). Moreover, compared with well-watered (WW) controls, the range of decrease in grain number was larger when WS occurred at YM stage rather than at anthesis. Thus, wheat at the YM stage was more sensitive to WS than at the anthesis stage, regarding grain number. Other research has also shown that reduction of grain number per spike was significantly larger as stress occurred before YM (<YM) rather than after YM (>YM) stage (Ji *et al.*, 2010). This was also observed in rice, where the time of highest sensitivity to stress coincided with the time of peak tapetal activity (YM stage; Oliver *et al.*, 2005).

Studies have shown that spike growth is directly affected by the stem and flag leaf, particularly under stress conditions. This in turn determines the number of fertile florets, and consequently, the final grain number during the critical period from the YM stage to anthesis (e.g. Fischer, 2011; Garcia *et al.*, 2014; Xie *et al.*, 2016). Whether the different responses of these three organs (spike, TIS and flag leaf) to WS during the two stages are associated with different degrees of grain abortion has not been elucidated in detail, particularly at the transcriptional level.

Here, transcriptional data revealed that the flag leaf, spike, and TIS had 2884, 970, and 382 DEGs, respectively, under WS at YM stage, while they had 39, 127, and 128 DEGs, respectively, under WS at anthesis (Fig. 3A-C). This suggests that more DEGs respond to WS at YM stage than at anthesis. The expression profile of three randomly selected genes were verified by qRT–PCR (Supplementary Fig. S1) to support the quality of RNA-seq data. A closer look at DEGs indicated that, compared with WS at YM stage, the number of DEGs under WS at anthesis were reduced by 98.7% (flag leaf), 79.5% (spike), and 24.8% (TIS; Fig. 3B, C). These findings indicated that the increased number of DEGs in organs in response to WS at YM stage may lead to the rapid reduction of grain number. This seems to be a conserved mechanism because plant organs showing rapid transcriptional responses are linked with a reduction of grain number under stress, which was also observed in soybean, rice and horse gram (Bhardwaj *et al.*, 2013; Fan *et al.*, 2013; Minh-Thu *et al.*, 2013; Sapeta *et al.*, 2016).

To analyse the function of the DEG, they were clustered into four groups (Fig. 3D) and annotated using GO enrichment tools (Fig. 3E, F). Clusters 1–3 revealed that the three organs (flag leaf, spike and TIS) maintained higher number of up-regulated genes (having function in signal transduction, response to abiotic stimuli, and protein metabolic processes) under WS at YM stage, than at anthesis [Fig. 3E(a-c)]. This suggests that as WS occurred at the YM stage, plants displayed increased gene expression via signal transduction to respond to abiotic stimuli. In cluster 4, the DEGs involved in carbohydrate metabolic processes were down-regulated, and the expression of genes was higher when WS occurred at anthesis, than at the YM stage [Fig. 3E(d)], indicating that carbohydrate metabolism may be more repressed by WS occurring at YM stage. Therefore, we suggest that although wheat has increased signal transduction when WS occurs at YM, lower carbohydrate metabolic capacity ultimately leads to a higher reduction in grain number, compared with WS occurring at anthesis. This finding was consistent with a previous study in which plants were reported to use a series of mechanisms to regulate reproductive growth to maximize plant fitness (Liu *et al.*, 2003).

### Competition-induced shortage of dry matter in the spike may result in a reduction of grain number

Grain number in wheat was identified as being the most sensitive to WS occurring at YM stage. Thus, the specific variation in superior (SGN) and inferior grain number (IGN) in each spikelet under WS occurring at YM stage was investigated. The reduction of grain number under WS was mainly attributed to a reduction of the SGN in the apical spikelet (15–20; Fig. 2A) and IGN in the central spikelet (7–14; Fig. 2B) in both cultivars. The IGN in the apical and basal spikelet was nearly completely aborted under WS. Moreover, only the IGN in the central spikelet, and both the IGN and SGN in the apical spikelet appeared to be affected by WS. Similar results have been previously recorded, i.e. that grain at the apical position within a spike and the farthest from the rachis was more infertile, particularly under stress conditions (González-Navarro *et al.*, 2015; Guo *et al.*, 2018).

Dreccer *et al.* (2009) focused on the trade-off between yield and grain number, which was closely related to dry matter accumulation (DMA), especially when resources were scarce under abiotic stress (Tardieu *et al.*, 2014). The present study also showed that DMA in the spike and the TIS was repressed by WS compared with WW controls, while a difference was not found in the flag leaf (Fig. 6A-C). This result indicated that DMA was more easily inhibited by WS in the spike and TIS, than in the flag leaf. This observation agreed with Hou *et al.* (2018), in that the DMA in stems was lower under stress. The rapid growth of spikes, stems, and leaves was inevitably accompanied by inequalities in dry matter partitioning (DMP) and even competition (Miralles *et al.*, 1998; Ferrante *et al.*, 2013). Here, the DMP increased markedly in the flag leaf but decreased in the spike under WS for the two cultivars, compared with the WW controls (Fig. 6D–F). Equally important, the DMA ratio between the spike versus flag leaf, and TIS versus flag leaf, was remarkably reduced compared to WW controls (Fig. 6H, I). These results suggested that fewer assimilates were transported into the spike and the TIS. To support this, the DMA ratio between the spike versus TIS was markedly reduced under WS (Fig. 6G), and a positive correlation between DMA and DMP was found in the TIS (Fig. 9B). Therefore, these results indicated that WS promoted the competitive capacity of TIS for assimilates by increasing DMA, which in turn led to poor spike fertility, and a subsequent reduction in the IGN and SGN at different spikelets. An increase in the DMA and DMP in the spike before anthesis would favor spike growth, and thus floret survival (Fischer, 2011; Garcia *et al.*, 2014; Xie *et al.*, 2016).

### Increased sucrose metabolism in the TIS leads to less sucrose in the spike under water stress

The competitive ability of organs with respect to assimilates influences grain number in crops (Marcelis, 1996; Marris, 2008). Assimilates are mainly accumulated in crops as carbohydrates, primarily consisting of sucrose, fructan and hexoses (fructose and glucose; Fischer, 1985; Xue *et al.*, 2008). In this study, because there was no difference between WS and WW treatments with respect to DMA in the flag leaf, the four major sugars and their respective enzyme activities were synchronously analysed only in the spike and the TIS. The results showed that WS resulted in a decrease in sucrose content in the TIS and spike (Fig. 7E, F), suggesting that the accumulation of sucrose was inhibited in both organs during WS. Hexose and fructan concentrations were increased in the TIS but inhibited in the spike when WS occurred at YM stage (Fig. 7A-D), indicating that sucrose was mainly cleaved into hexose and fructan in the TIS. In addition, reduced sucrose in the spike may be due to the strong ability of the TIS to cleave sucrose, which leads to an insufficient sucrose supply from the TIS. These processes in turn would determine spike fertility and final grain abortion. This finding is consistent with Wang *et al.* (2015), who reported that sucrose was transported long distances from the source leaf through the stem to the spike for grain and fruit set. If the sucrose supply is decreased in the spike, or the hydrolysis of sucrose into hexoses is weak, grain formation will be interrupted (McLaughlin and Boyer, 2004; Hiyane *et al.*, 2010; Shen *et al.*, 2018). Only hexose was significantly and negatively correlated with DMA in the TIS and spike, indicating that the ability of sucrose to convert into hexose was a consequence of reduced DMA in this study.

In fact, sucrose metabolism and transportation in plants are regulated by a series of enzymes (Valluru *et al.*, 2008; Ruan *et al.*, 2014). Invertases, including acid-insoluble or cell wall-bound inverstase (CWIN) and acid-soluble vacuolar invertase (VIN), hydrolyse sucrose into hexose according to their optimum pH and sub-cellular locations. Here, an enzyme assay revealed that only CWIN was induced by WS in the TIS and spike (Fig. 8A-D), suggesting that sucrose may be primarily unloaded and utilized. CWIN in plant sink organs is responsible for sucrose phloem unloading and subsequent utilization (Ruan *et al.*, 2012; Rottmann *et al.*, 2018). Simultaneously, fructan is regulated by biosynthetic (1-SST) and hydrolytic (1-FEH) enzymes (Van den Ende *et al.*, 2006). In this study, only 1-SST activity was induced in the TIS in response to WS. Moreover, 1-SST showed a positive correlation with fructan concentrations in the TIS (Fig. 9G). Thus, these results suggested that sucrose was rapidly unloaded and then degraded into hexose by CWIN, and into fructan by 1-SST, in the TIS as a response to stress. Fructan is an important product of sucrose metabolism in stems under stress conditions (Chen *et al.*, 2015). In the spike, although an increase of CWIN and 1-SST activities were observed (Fig. 8C, E), all sugars were reduced (Fig. 7A, C, E, G). The spike had the highest gene expression in response to abiotic signals, compared with the TIS and flag leaf at the YM stage [Fig. 3E(a)]. These results suggested that the activity of these enzymes (1-SST and CWIN) may be increased in response to abiotic stimuli, rather than sucrose metabolism. CWIN has been shown to interact with other signaling pathways independent of sugars, and help control grain growth (Muller *et al.*, 2011).

**ABA concentration and signal transduction participate in sucrose metabolism**

ABA and IAA regulate plant growth and participate in responses to abiotic stresses, including drought (Rahman, 2013). ABA and IAA synthesis and signal transduction may ultimately regulate sugar through enzymes (Li *et al.*, 2017). In this study, the concentration of IAA [Fig. 9A, B] and the expression of genes involved in IAA signal transduction (Fig. 4B) were repressed by WS in the spike and TIS. Moreover, both organs showed similar expression of genes involved in IAA signal transduction. The IAA/ABA ratios in the spike and the TIS were also reduced by WS. IAA and IAA/ABA showed a positive correlation with DMA in both organs. These results suggested that the growth of the spike and TIS were inhibited by WS. The concentration of ABA (Fig. 9C, D) was markedly increased, while the expression of genes involved in ABA signal transduction was inhibited by WS in the spike and TIS (Fig. 4A). The TIS showed higher expression of genes involved in ABA signal transduction compared with the spike. In addition, the ABA concentration showed a significant positive correlation with 1-SST and CWIN in the TIS (Fig. 9G). Collectively, the higher down-regulation of ABA signal transduction-related genes in the TIS than the spike may promote sucrose hydrolysis in the TIS despite an increase in ABA in both organs, finally leading to a weak sucrose supply in the spike. Other stressors had a similar result, and dynamic transcriptional changes were induced by stress signals, followed by the activation of metabolic processes (Ikeuchi *et al.*, 2017). In land plants, ABA plays a vital role in the regulation of key enzymes involved in sucrose metabolism in wheat (Yang *et al.*, 2004; Kobayashi *et al.*, 2005; Szemenyei *et al.*, 2008). Therefore, ABA induced an increase of 1-SST activity, which promoted sucrose to synthesize into fructan in the TIS.

**Fig. 10.**
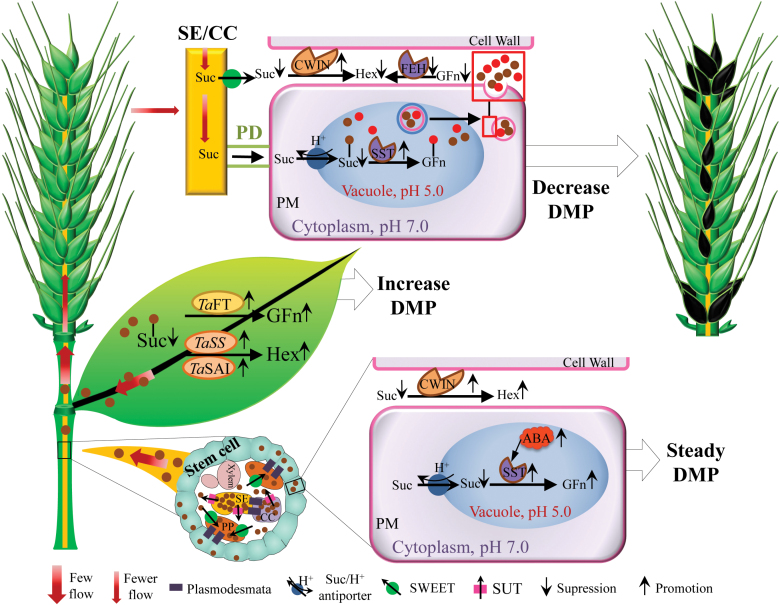
Sucrose (Suc) is unloaded, transported, and metabolized in plant organs under water stress. Suc is rapidly degraded into hexose (Hex) and fructan (GFn) in flag leaf under water stress (WS) at the YM stage, which results in an increase of dry matter partitioning (DMP). Thus, less Suc is transported through the sieve element/companion cell complex (SE/CC) to non-photosynthetic tissues, such as stems and spikes. ABA induces conversion of Suc into Hex by cell wall invertase (CWIN), before being taken up into the cytoplasm and GFn by sucrose:sucrose 1-fructosyl-transferase (1-SST) in the vacuole, leading to steady DMP of the two-internode under spike. Less Suc is transported into spike and consequently, reduces Hex and GFn. Correspondingly, a decrease of DMP is observed in the spike. Collectively, superior grains at the apical and basal spikelet and inferior grains at the central spikelet were aborted. Black grains represent aborted grains. PD, plasmodesmata; PP, phloem parenchyma; PM, plasma membrane; SWEET and SUT, sucrose transporter. (This figure is available in colour at *JXB* online.)

Overall, we propose a mechanism to describe the reduction of grain number caused by the coordination in flag leaf, TIS and spike at physiological and transcriptional levels (Fig. 10). Under water stress, the up-regulation of soluble acid invertase (*Ta*SAI), sucrose synthase (*Ta*SS), vacuole invertase (*Ta*VIN1) and fructosyl transferase (*Ta*FTase) in the flag leaf metabolizes sucrose into hexose and fructan, maintaining a steady DMA level and lesser export of sucrose. In the TIS and spike, ABA synthesis, CWIN and 1-SST is increased, but the down-regulation of ABA signal transduction related-genes in the TIS is higher than that in the spike. Correspondingly, sucrose is unloaded and degraded into hexose and fructan more in the TIS, than in the spike, which contributed to a higher DMP in the TIS than in the spike. Therefore, the co-ordination and different metabolic responses of these organs finally results in a decrease of grain number under WS.

## Supplementary data

The following supplementary data are available at *JXB* online.

Table S1. Sequencing and assembly statistics for the transcriptional data of the spike, two-internode under spike (TIS) and flag leaf during the young microspore (YM) stage and anthesis under well-watered (WW) and water stress (WS) treatments.

Table S2. Primer list used to confirm gene expression (qRT–PCR validation).

Table S3. Differentially expressed genes were found in the spike, two-internode under spike (TIS) and flag leaf of winter wheat during the young microspore stage (YM) under water stress.

Table S4. Differentially expressed genes were found in the spike, two-internode under spike (TIS) and flag leaf of winter wheat during anthesis in plants under water stress.

Table S5. The cluster 1 of DEGs by *k*-means analysis.

Table S6. The cluster 2 of DEGs by *k*-means analysis.

Table S7. The cluster 3 of DEGs by *k*-means analysis.

Table S8. The cluster 4 of DEGs by *k*-means analysis.

Table S9. Gene Ontology (GO) enrichment analysis of differentially expressed genes conducted in the spike, two-internode under spike (TIS) and flag leaf during the young microspore (YM) and anthesis stages under water stress.

Table S10. Metabolic pathway analysis of differentially expressed genes in the spike, two-internode under spike (TIS) and flag leaf during the young microspore (YM) and anthesis stages under water stress.

Table S11. The cluster 1 of DEGs by *k*-means analysis with corresponding GO enrichment.

Table S12. The cluster 2 of DEGs by *k*-means analysis with corresponding GO enrichment.

Table S13. The cluster 3 of DEGs by *k*-means analysis with corresponding GO enrichment.

Table S14. The cluster 4 of DEGs by *k*-means analysis with corresponding GO enrichment.

Figure S1. Expression of three genes inferred by RNA-sequencing and qRT–PCR.

eraa380_suppl_Supplementary_Figure_S1

eraa380_suppl_Supplementary_Table_S1-S14

## Data Availability

The data that support the findings of this study are available from the corresponding authors, upon request.
