# Supplementary figures and images for: Suppressed ABA signal transduction in the spike promotes sucrose use in the stem and reduces grain number in wheat under water stress
Source: J Exp Bot. 2020 Aug 21;71(22):7241–56. doi: 10.1093/jxb/eraa380 (PMC7906786; doi:10.1093/jxb/eraa380)

RNA-seq Expr. (FPKM)

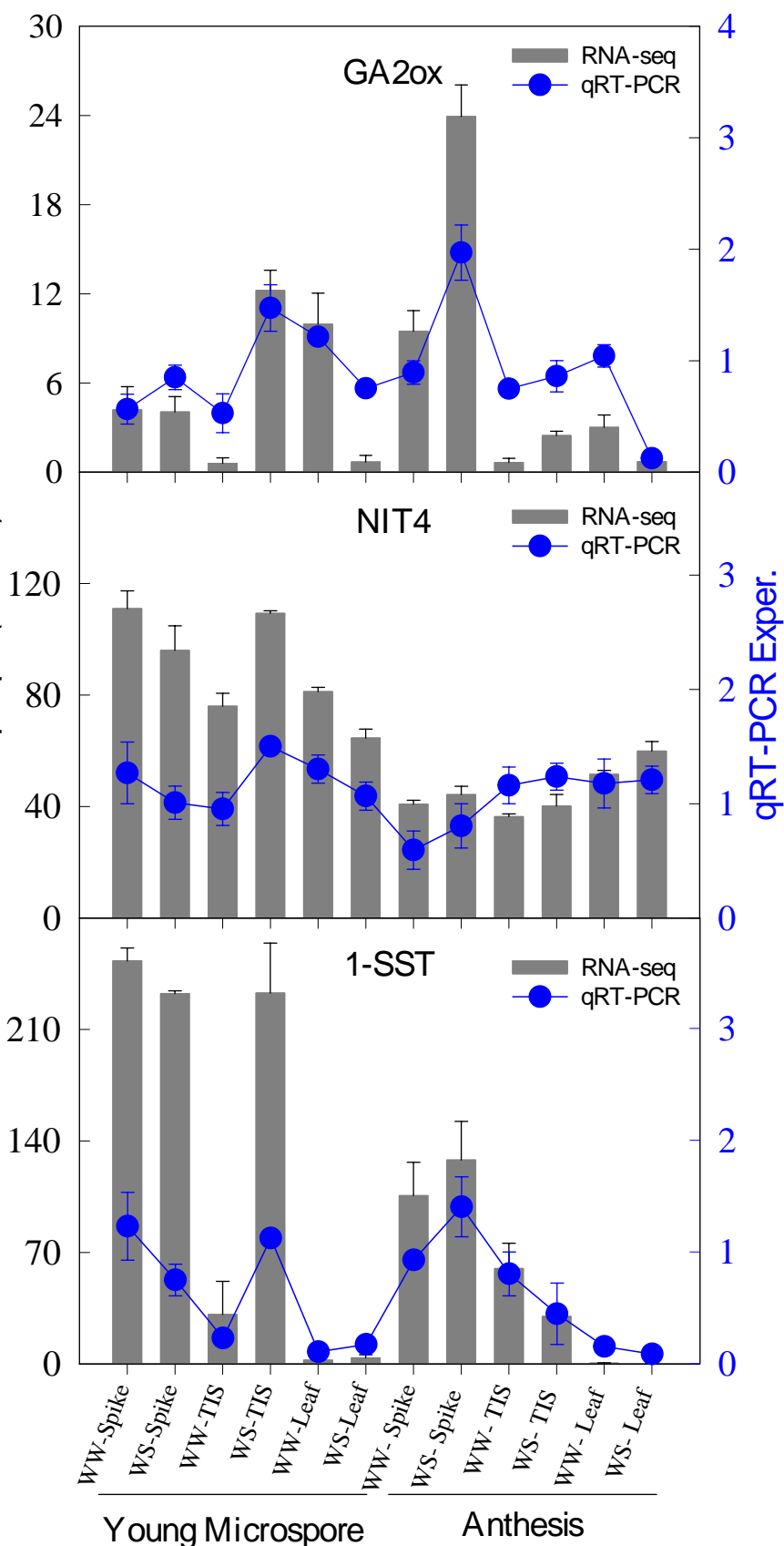

Supplement: eraa380_suppl_Supplementary_Figure_S1 [file eraa380_suppl_supplementary_figure_s1.pdf]
